# Study on Differences in Structure and Anti-Inflammatory Activity of Polysaccharides in Five Species of Dendrobium

**DOI:** 10.3390/polym17091164

**Published:** 2025-04-24

**Authors:** Hua Zhu, Hui-Wen Zhang, Jia-Hao Fan, Si-Si Jia, Xin Yi, Zi-Wei Han, Ren-Lei Wang, Hong-Wei Qiu, Guang-Ping Lv

**Affiliations:** 1 School of Life Science, Nanjing Normal University, Nanjing 210046, China; wfzh8233@163.com; 2 School of Food and Pharmaceutical Engineering, Nanjing Normal University, Nanjing 210046, China; okeydokey949@163.com (H.-W.Z.); fanjiahao1203@163.com (J.-H.F.); 18835540923@163.com (S.-S.J.); 212702035@njnu.edu.cn (X.Y.); hinziwei@163.com (Z.-W.H.); 3 Key Laboratory of Innovative Applications of Bioresources and Functional Molecules of Jiangsu Province, College of Life Science and Chemistry, Jiangsu Second Normal University, Nanjing 211200, China; wrl@jssnu.edu.cn

**Keywords:** Dendrobium, polysaccharides, saccharide mapping, anti-inflammatory activity

## Abstract

Dendrobium is a famous edible and medicinal plants, and polysaccharides are their main bioactive components. Polysaccharides from five species, namely, DO (*Dendrobium officinale* Kimura et Migo), DH (*Dendrobium huoshanense* C. Z. Tang et S. J. Cheng), DNL (*Dendrobium nobile* Lindl.), DFH (*Dendrobium fimbriatum* Hook.), and DCL (*Dendrobium chrysanthum* Lindl.), were compared based on molecular weight (Mw), monosaccharide composition, and glycosidic bond types. The results showed that *Dendrobium* polysaccharides (DPs) contain relatively simple compositional monosaccharides and mainly consist of mannose (Man) and glucose (Glc), along with small amounts of arabinose (Ara), xylose (Xyl), and galactose (Gal). The Am/Ag (the ratio of Man to Glc) values in DO, DH, and DNL polysaccharides were 3.23, 3.81, and 3.88, while those in DFH and DCL were 0.45 and 0.81. DPs are mainly composed of →4)Man*p*(1→ and →4)Glc*p*(1→, but their molar ratios were different. →4)Man*p*(1→ and →4)Glc*p*(1→ ratios were 2.85, 2.92, 1.50, 1.45, and 1.05 in DO, DH, DNL, DFH, and DCL, respectively. Hierarchical cluster analysis (HCA) showed that there were significant differences in structural information, especially in glycosidic bond types and proportions. DH, DO, and DCL were clustered into different groups based on glycosidic bond types and proportions, respectively. Moreover, the five species of Dendrobium could significantly inhibit NO production and apoptosis induced by LPS in RAW 264.7, especially DH. The results of a correlation analysis of structure and anti-inflammatory activity showed that polysaccharides with a high →4)Man*p*(1→/→4)Glc*p*(1→ ratio and a molecular weight distribution between 3.343 × 10^5^ Da and 13.540 × 10^5^ Da had better anti-inflammatory activity. The results indicated that the quality evaluation of Dendrobium in clinical applications should investigate molecular weight and the composition of the glycoside bond types and proportions to ensure the consistency of curative effects.

## 1. Introduction

Dendrobium is a well-known edible and medicinal herb worldwide. However, there are many different species in the genus, and their characteristics vary greatly [1]. The 2020 edition of the Chinese Pharmacopoeia documented five Dendrobium species including *D. officinale* Lindl. (DO), *D. huoshanense* C. Z. Tang et S. J. Cheng (DH), *D. fimbriatum* Hook. (DFH), *D. nobile* Lindl. (DN), and *D. chrysanthum* Lindl (DCL). Dendrobium contains polysaccharides, phenols, alkaloids, and other active ingredients. Dendrobium species are known for their many pharmacological effects, such as anti-tumor, anti-fatigue, antioxidant, anti-aging, anti-inflammatory, liver protection, immunomodulatory, and other pharmacological effects [2].

Currently, the exploitation and utilization of Dendrobium plant resources have mainly focused on water-soluble polysaccharides. Indeed, Dendrobium polysaccharides have attracted much attention in recent years due to their various pharmaceutical activities, such as anticancer, anti-hyperglycemic, anti-fatigue, anti-inflammatory, and immunomodulatory activities [3,4,5,6]. The bioactivity of polysaccharides is usually not only related to content, but also related to structural characteristics. In fact, the biological activity of polysaccharides is closely related to their chemical properties, such as the molecular weight, type and proportion of monosaccharides, and characteristics of glycosidic bonds [7]. Previous reports indicated that polysaccharides from four commonly used *Polygonatum* spp. differed in their monosaccharide composition and high-performance gel permeation chromatography (HPGPC) saccharide mapping [7]. In a previous study, polysaccharides from DH were primarily composed of mannose and glucose, with molecular weights ranging from 1.16 × 10^5^ to 2.17 × 10^5^ Da and main glycosidic linkages of →4)Glc*p*(1→ and →4)Man*p*(1→, substituted with acetyl groups at O-2 and O-3 of →4)Man*p*(1→ [8]. It is worth noting that there are many Dendrobium varieties on the market at present, among which DH and DO are often forged due to their high prices. This phenomenon has seriously affected the development of the Dendrobium industry. However, an overall picture of the chemical composition of polysaccharides in Dendrobium species has not been elucidated. The diverse chemical characteristics of the polysaccharides from Dendrobium (DPs) may be correlated with their health benefits. Unfortunately, there are few reports in this field.

In this study, five polysaccharides from Dendrobium (DO, DH, DNL, DFH, and DCL) were extracted, and the molecular weight distribution, monosaccharide composition, and glycosidic bond types were systematically characterized. Furthermore, structural information on Dendrobium polysaccharides was analyzed by the hierarchical cluster analysis (HCA) method. Finally, this study evaluated the anti-inflammatory potential of various Dendrobium polysaccharides by measuring their ability to inhibit NO production and reduce apoptosis in RAW 264.7 cells. These methods clarify the structural association of Dendrobium polysaccharides with anti-inflammatory activity, analyze polysaccharide structures that may affect anti-inflammatory activity, further compare the anti-inflammatory activity of five Dendrobium species, and investigate their regulatory effects on apoptosis, which have not been elucidated previously. This study provides a basis for the quality evaluation of Dendrobium polysaccharides, and also provides a reference for the development of functional or nutritional products of Dendrobium polysaccharides.

## 2. Materials and Methods

### 2.1. Materials and Reagents

Five species of Dendrobium were collected from different regions of China (Table 1). All samples were deposited at the Nanjing Normal University (Nanjing, Jiangsu, China). Monosaccharide standards of mannose (Man), glucose (Glc), galactose (Gal), ribose (Rib), arabinose (Ara), xylose (Xyl), rhamnose (Rha), and fructose (Fru) were acquired from Sigma-Aldrich (St. Louis, MO, USA). Neutral red was obtained from Shanghai solarbio Bioscience & Technology Co. (Beijing, China). The NO assay kit and apoptosis and necrosis assay kit were obtained from Beyotime Biotechnology Co., Ltd. (Shanghai, China). All other chemicals were of analytical grade and purchased from Sinopharm Chemical Reagent Co., Ltd. (Shanghai, China).

### 2.2. Extraction of Polysaccharides

The dried sample was powdered. The powdered sample was soaked in 60% ethanol for 2 h with stirring, to remove pigments and small organic compounds. After filtration and drying, the residues were extracted with boiling water for 2 h. The filtrates were concentrated to about 10 mL, and then precipitated using 80% (*v*/*v*) ethanol and freeze-drying to obtain the polysaccharides of DO, DH, DNL, DFH, and DCL.

### 2.3. Characterization of Polysaccharides from DPs

#### 2.3.1. Molecular Weight (Mw) Distribution Analysis

The molecular weights of the DPs from different sources were evaluated by high-performance size-exclusion chromatography (HPSEC), combined with multi-angle laser light scattering system (MALLS, Dawn Heleos Wyatt Technology Co., Santa Barbara, CA, USA) and a refractive index detector (RID, Shimadzu Global Laboratory Consumables Co., Ltd., Tokyo, Japan) [8]. The columns of the SB-806HQ (8.0 mm × 300 mm) and SB-804HQ (8.0 mm × 300 mm, Showa Denko KK, Tokyo, Japan) were used for separation at 35 °C. The detailed experimental conditions were as follows: DP powder (1.5 mg) was dissolved in 1 mL NaCl (0.9%); the column and RID temperatures were maintained at 35 °C; the injection volume was 200 μL; and the samples were eluted with 0.9% NaCl at a flow rate of 0.5 mL/min. The refractive index increment (*dn*/*dc*) was set to 0.15 mL/g. The data collected by HPSEC-MALLS-RID were then analyzed using ASTRA 5.34 software (Wyatt Technology, Santa Barbara, CA, USA) [9].

#### 2.3.2. Monosaccharide Compositional Analysis

The monosaccharide composition was determined according to our previous report [9]. First, 3 mg was mixed with an equal volume of 2 mol/L trifluoroacetic acid (TFA) and hydrolyzed at 90 °C for 6 h. The hydrolyzed product was dried with N_2_, and then the residue was redissolved with methanol. Then, split into two, the ketones and aldehydes were subjected to monosaccharide derivatization separately. The monosaccharide derivatization products were determined by GC-MS (Shimadzu QP2020 NX, Shimadzu Global Laboratory Consumables Co., Ltd., Tokyo, Japan) using HP-5MS (30 m × 0.25 mm ID, 0.25 μm film thickness, Agilent Technologies Inc., Santa Clara, CA, USA).

#### 2.3.3. Methylation Analysis

Based on previous reports, the DPs could be fully methylated [10]. DPs (5 mg) were dissolved with 1 mL of dimethyl sulfoxide (DMSO) and then combined with NaOH (20 mg) and ultrasound for 60 min. Afterward, CH_3_I was added and ultrasound-incubated for 60 min, and the above steps were repeated two times. The partially methylated DPs were hydrolyzed for 30 min with 0.5 mol/L TFA and then dried. Following a 2 h reduction with NaBH_4_, the monosaccharide underwent subsequent treatment with acetic anhydride and pyridine for 30 min to convert the released monosaccharides to partially methylated alditol acetate, which was finally analyzed by GC-MS. The temperature conditions were set as follows: the initial temperature of the column was 140 °C, the temperature was increased to 150 °C at 5 °C/min, then from 150 °C to 200 °C at an increment of 10 °C/min, and from 200 °C to 230 °C at 2 °C/min, and finally the temperature was increased to 300 °C and held for 5 min. The injection port temperature was set at 250 °C, and the carrier gas was high-purity helium. Partially methylated alditol acetates (PMAAs) were identified by their fragment ions according to the MS spectrum and the relative retention time on the GC profile.

### 2.4. Effects of Polysaccharides on Macrophage Functions

#### 2.4.1. Cell Culture

RAW 264.7 cells were obtained from Shanghai Life Science Research Institute Cell Resource Center (Shanghai, China). Cells were cultured in Dulbecco’s Modified Eagle Medium (DMEM) supplemented with 10% Fetal Bovine Serum (FBS) and 1% penicillin/streptomycin at 37 °C in a humidified atmosphere of 5% CO_2_.

#### 2.4.2. Cell Viability Assay

RAW 264.7 cells were seeded in cell culture plates and cultured for 24 h in medium containing DPs (0, 5, 10, 20, 50, 100, 200, 400 μg/L). Meanwhile, an equal volume of culture medium was used as a control. Subsequently, 10% CCK-8 reagent was added for incubation for 3 h, and the optical density at 450 nm was measured. Cell viability was calculated as the ratio of absorbance values between sample and control groups [11].

#### 2.4.3. NO Assay

RAW 264.7 macrophages were inoculated in 96-well microtiter plates at 3 × 10^5^ cells/well. The plates were incubated with DPs (0, 10, 50, 200 μg/mL) and LPS (1.0 μg/mL) for 24 h [8]. The NO content of RAW 264.7 cells was determined by the NO assay kit (Beyotime Biotechnology Co., Ltd., Shanghai, China). Briefly, the plates were mixed with an equal volume of Griess reagent, and the absorbance was measured at 540 nm.

#### 2.4.4. Measurements of Apoptosis

The cell apoptosis and necrosis assay kit was used for the detection of apoptosis in RAW 264.7 cells. Briefly, RAW 264.7 cells were inoculated into DPs (0, 10, 50, 200 μg/mL), seeded in 96-well plates (with or without LPS treatment), incubated for 24 h, and centrifuged to collect the supernatant, and cell apoptosis was quantified by the kit. The BD Accuri™ C6 Cytometer (BD Biosciences, San Jose, CA, USA) was used for analysis.

### 2.5. Statistical Analysis

For multiple group comparisons, one-way analysis of variance (ANOVA) was conducted by Duncan’s post hoc test and applied for determining the significance (*p* < 0.05) of differences. Other statistical analysis was carried out using SPSS software (version 18.0, SPSS Inc., New York, NY, USA.), including HCA for similarity display. Gray relational analysis was conducted using SPSSPRO (http://www.spsspro.com (accessed on 14 April 2025)).

## 3. Results and Discussion

### 3.1. Molecular Weight (Mw) Distribution Analysis of DPs

**Figure 1:**
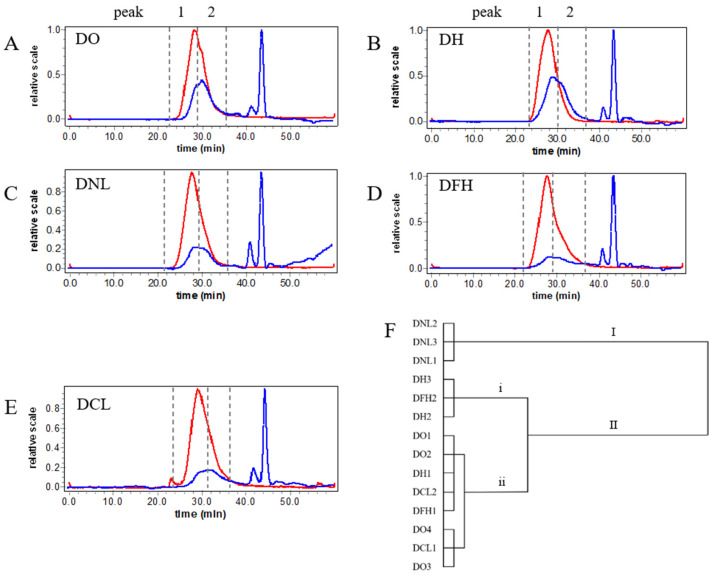
HPSEC-MALLS-RID chromatograms of polysaccharides from different species of Dendrobium: DO (**A**), DH (**B**), DNL (**C**), DFH (**D**), and DCL (**E**). Dendrogram for hierarchical cluster analysis, with Furthest neighbor selected as measurement for molecular weight distribution (**F**). The red line represents the signal from the RID, and the blue line represents the signal from the MALLS. I, II, i and ii stand for classification level.

HPSEC-MALLS-RID chromatograms demonstrated that the molecular weight distribution of polysaccharides is a powerful tool for discrimination and improvement in the quality evaluation of polysaccharides from natural sources in previous studies [12,13]. Usually, the biological activity of natural polysaccharides is affected by their molecular weight distribution. Therefore, the molecular weight distributions of DPs from different species in China were measured with HPSEC-MALL-RID. Figure 1A–E show representative HPSEC-MALL-RID chromatograms of DPs from five species of Dendrobium in China. In Figure 1, RID signals (blue) show that there are two fractions with similar molecular weights (Peak 1–2) in five species of Dendrobium, and the MALLS signals (red) show the properties of their molecules. Importantly, the HPSEC chromatograms of polysaccharides from different species of Dendrobium in China were different.

The Mw calculated by the HPSEC-MALL-RID system is shown in Table 2; there is similarity in the molecular weights of the same species of Dendrobium, such as DNL and DCL, but there is some variability in the mean molecular weights of different species of Dendrobium (*p* < 0.05). The average molecular weight of Peak 1 of DFH (10.027 ± 3.848 × 10^5^ Da) was 1.26, 1.74, 1.86, and 2.99 times higher than that of DO (7.987 ± 3.035 × 10^5^ Da), DH (5.750 ± 1.673 × 10^5^ Da), DNL (5.388 ± 0.441 × 10^5^ Da), and DCL (3.354 ± 0.054 × 10^5^ Da), respectively. The average molecular weight of Peak 2 of DFH (3.300 ± 1.819 × 10^5^ Da) was 1.71, 1.99, 2.23, and 4.65 times that of DO (1.204 ± 0.063 × 10^5^ Da), DH (1.660 ± 0.091 × 10^5^ Da), DNL (1.479 ± 0.156 × 10^5^ Da), and DCL (0.709 ± 0.166 × 10^5^ Da), respectively. The Mw of the polysaccharide in DCL is significantly different from that of DFH. In the comparison of the Mw of polysaccharides of DCL and DFH in Table 2, it is evident that the molecular weights of DCL and DFH at Peak 1 and Peak 2 are significantly different (*p* < 0.05). Peak 1 and Peak 2 of DFH are both larger than DCL. The biggest difference is in Peak 2, which is about 4.65 times higher than DCL. The polydispersity index (PDI) of Dendrobium polysaccharide Peak 1 ranges from 1.085 ± 0.046 to 1.622 ± 0.039. The PDI for Peak 2 ranges from 1.047 ± 0.042 to 1.642 ± 0.030. The difference in molecular weight distribution between DCL and DFH polysaccharides may be due to mannan chain length and the degree of branching [14].

In addition, a hierarchical cluster analysis of 14 samples was performed based on the molecular weight distribution data. Samples in a dataset can be hierarchically clustered in order to discover similarities between the samples. Similarity evaluation methods play a crucial role in HCA because they determine how similarity between samples is measured and calculated. Therefore, samples with similar characteristics are clustered in the same class; on other hand, samples divided into different groups have different characteristics. In the HCA dendrogram (Figure 1F), it can be seen that DNL is clustered in Class I. DO, DH, DFH, and DCL are clustered in Class II, where DO and DCL were clustered in Class II (ii). The results show that the information on molecular weight was insufficient to achieve the discrimination of different Dendrobium species. Therefore, monosaccharide composition and the primary chemical structure should be further clarified.

### 3.2. Monosaccharide Compositions of DPs

**Figure 2:**
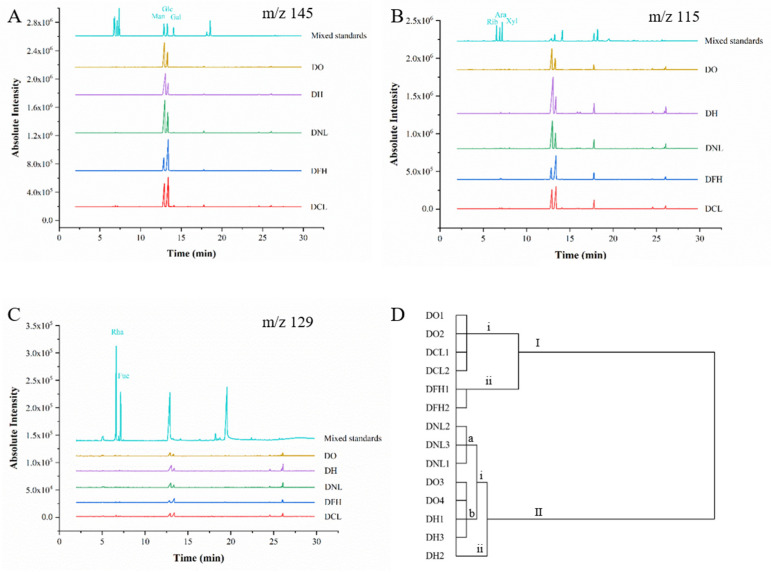
GC-MS chromatograms of extracted ion at *m*/*z* 145 (**A**), 115 (**B**), and 129 (**C**) of standard monosaccharides and hydrolysis products of DO, DH, DNL, DFH, and DCL. Dendrogram for hierarchical cluster analysis of 14 tested samples, with Euclidean distance selected as measurement for compositional monosaccharides (**D**). I, II, i, ii, a and b stand for classification level.

GC-MS analysis has been widely applied to the qualitative and quantitative analysis of compositional monosaccharides in polysaccharides from medicinal plants [8,10]. Monosaccharides are the basic units of polysaccharides, which vary significantly with plant species [15]. Figure 2A–C and Table 3 show representative GC-MS chromatograms of compositional monosaccharides from five species of Dendrobium in China. The results show that DPs mainly consisted of Man and Glc with very small amounts of Ara, Xyl, and Gal. These findings are consistent with those of a previous study demonstrating that the main polysaccharide fractions in Dendrobium were composed of Man and Glc [16,17]. However, the ratio of Man to Glc (Am/Ag) varies between different Dendrobium species. As shown in Table 3, DO, DH, and DNL mainly contain Man, DFH mainly contains Glc, and the contents of Man and Glc in DCL are similar. The Am/Ag ranges of DO, DH, DNL, DFH, and DCL are 2.23–4.22, 3.40–4.49, 2.75–4.76, 0.42–0.48, and 0.76–0.86. In all samples, DNL has the highest Am/Ag average value of 3.88. For DH, DO, DCL, and DFH, the Am/Ag average value was 3.81, 3.23, 0.81, and 0.45. The results showed that the Am/Ag values of DO, DH, and DNL polysaccharides were greater than 2, while those of DFH and DCL polysaccharides were less than 1. In addition, according to the specification of *Dendrobium officinale* (DO) in the China Pharmacopoeia 2020 edition, the ratio of Man to Glc in polysaccharides from DO should be in the range of 2.4 to 8.0. All samples of DO are in this range in this study. It was reported that Am/Ag in polysaccharides may be related to activity [18]. For DH, which is considered the most effective species of Dendrobium, Am/Ag is also in this range [8,19]. However, for DFH and DCL, which are documented as sources of Dendrobium, the Am/Ag values are not in the range of 2.4 to 8.0. This indicates that more structural information should be obtained and bioactivity evaluation carried out to investigate the differences in different species of Dendrobium.

In addition, HCA was also performed based on the monosaccharide compositions of DPs. As shown in Figure 2D, all samples were classified into two clusters; it can be seen that DCL and DFH are clustered in Class I, for which Am/Ag is less than 1 (Table 3).

DNL, with the highest average Am/Ag, is also separated in class II (i) a. DO and DH, which have similar Am/Ag values, are not separated. The results show that there are differences in monosaccharide compositions among the five species of Dendrobium. These differences in monosaccharide composition between different species of DPs imply different structural features of the polysaccharides, which may play an important role in specific structure–activity relationships. The monosaccharide composition and Am/Ag of polysaccharides are very important for understanding the structure characterizations of different Dendrobium species, and can be used as an important reference for evaluating the quality and consistency of Dendrobium used in clinical contexts. However, information on monosaccharide composition was insufficient for evaluations due to unknown structural properties, such as the DO and DH with similar Am/Ag values. Therefore, the primary chemical structure should be further elucidated.

### 3.3. Glycosidic Linkages of DPs

**Figure 3:**
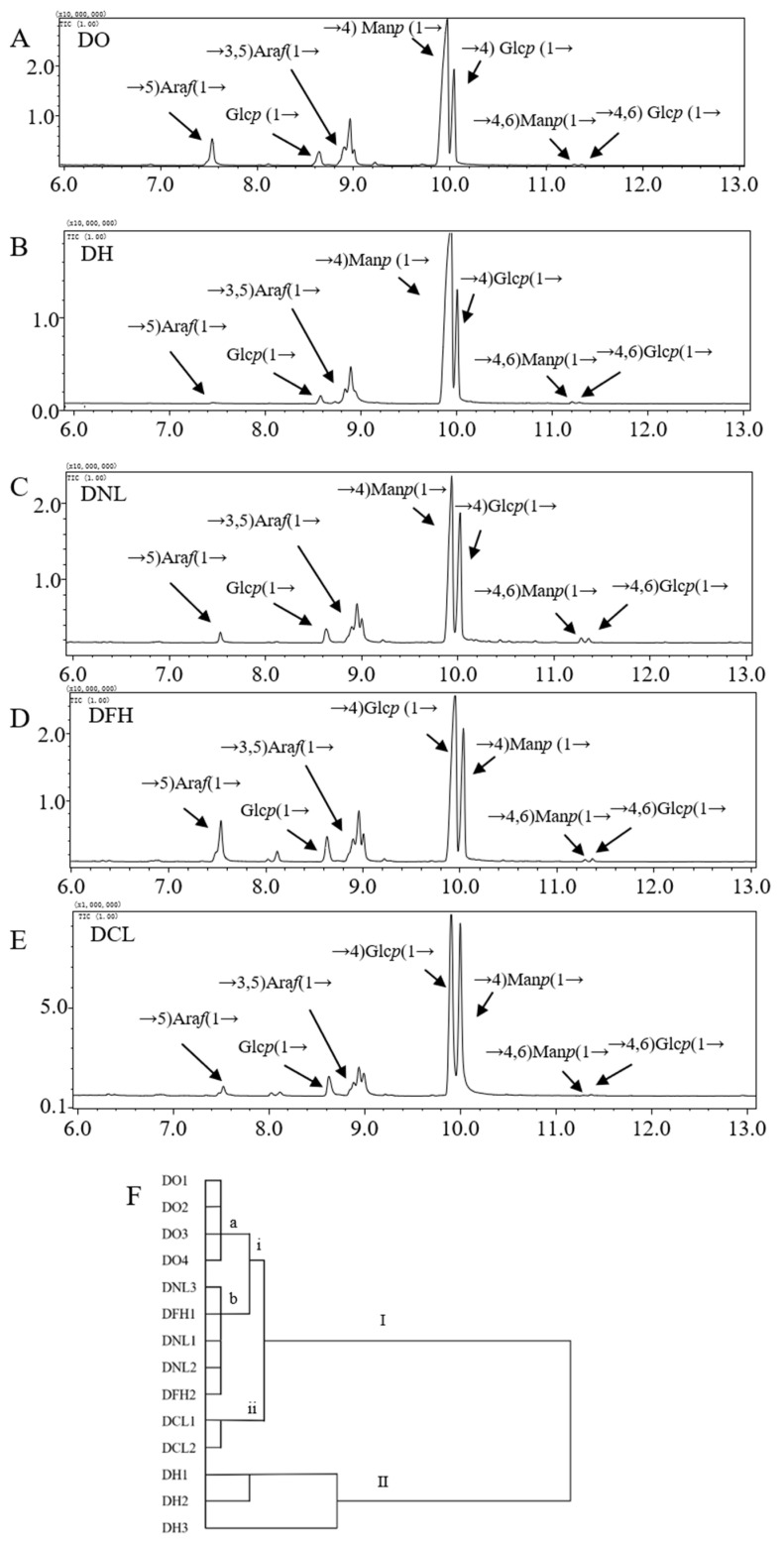
GC-MS total ion chromatography of partially methylated alditol acetate of polysaccharides from different species of Dendrobium: DO (**A**), DH (**B**), DNL (**C**), DFH (**D**), and DCL (**E**). Dendrogram for hierarchical cluster analysis of 14 tested samples, with Chebyshev distance selected as measurement for glycosidic bond types (**F**). I, II, i, ii, a and b stand for classification level.

Methylation analysis is traditionally applied for providing information on the glycosidic linkages and branches in polysaccharides from medicinal plants [18,20]. Representative chromatograms of DPs and types and proportions of glycosidic bonds are shown in Figure 3A–E. The results show that the main linkage types in the five species of Dendrobium polysaccharides (DO, DH, DNL, DFH, and DCL) were →4)Man*p*(1→ and →4)Glc*p*(1→, as shown in Table 4. It was found that the ratios of →4)Man*p*(1→ and →4) Glc*p*(1→ in DO, DH, DNL, DFH, and DCL were 2.85, 2.92, 1.50, 1.45, and 1.05, respectively. Also, DPs contain small amounts of Ara*f*(1→, →5)Ara*f*(1→, Glc*p*(1→,→3,5) Ara*f*(1→, →4,6)Man*p*(1→, and →4,6)Glc*p*(1→. These results suggest that the polysaccharides in Dendrobium had a backbone consisting of →4)Man*p*(1→ and →4)Glc*p*(1→, and there are substituents at O-6 site of branch point→4,6)Man*p*(1→ and →4,6)Glc*p*(1→, which is in accordance with a previous study [21].

To further evaluate the differences between different Dendrobium species, HCA was performed according to the glycosidic bond type and content of polysaccharides (Figure 3F). As shown in Figure 3F, DO, DCL, and DH polysaccharides were clustered in separate groups (Class I (i) a, I (ii), and II). It can be seen that the glycosidic bond types and ratios are particularly important for the quality evaluation of Dendrobium polysaccharides. The type and proportion of glycosidic bonds of polysaccharides have a significant effect on the pharmacological activity of polysaccharides, and this characteristic showed differences in different Dendrobium species, indicating that it can be used as an effective means for the discrimination and evaluation of polysaccharides from natural sources [8].

### 3.4. Effects of DPs on Macrophage Functions

Polysaccharides are one of the most important bioactive compounds in Dendrobium, especially in terms of anti-inflammatory activity. For polysaccharides in natural products, there is a close relationship between biological activity and their chemical properties, such as the molecular size, type and proportion of compositional monosaccharides, and characteristics of glycosidic bonds [17]. In this study, the chemical properties of DPs were systematically investigated, including molecular weight distribution, monosaccharide composition, and glycosidic bonds. In addition, RAW 264.7 induced by LPS was used to evaluate the anti-inflammatory effect of DPs in vitro in order to better understand the structure–activity relationship of DPs.

#### 3.4.1. Effects of DPs on Cell Survival

**Figure 4:**
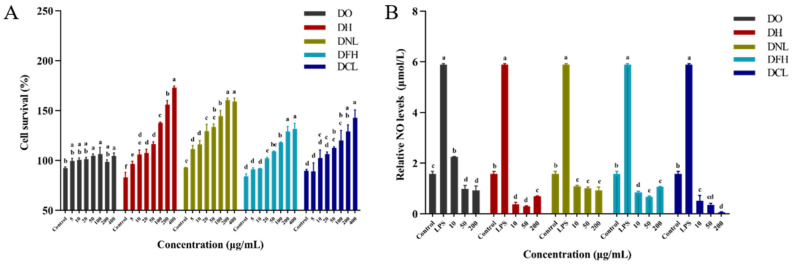
Anti-inflammatory effect of DPs evaluated by RAW 264.7 macrophages. Cell survival (**A**) and NO production (**B**) of DO, DH, DNL, DFH, and DCL polysaccharides. Values are presented as means ± SD (*n* = 3). a–f—Different letters indicate significant differences (*p* < 0.05).

The effects of varying concentrations of Dendrobium polysaccharides (DPs, 5–400 μg/mL) on RAW 264.7 macrophage viability were assessed using the CCK-8 assay. This assay relies on the reduction of CCK-8 by mitochondrial dehydrogenases in viable cells, producing a water-soluble orange formazan product. Absorbance (OD) was measured at 450 nm, with values directly proportional to cell proliferation and inversely proportional to cytotoxicity. As shown in Figure 4A, the results of this study reveal that all five DPs had no cytotoxicity against RAW 264.7 cells. Among them, DH, DNL, DFH, and DCL promoted macrophage proliferation significantly, and the extent of their promotion effect depended on the dose in a specific concentration range, whereas DO has no significant cell proliferation effects at 5–20 μg/mL. At 400 μg/mL, the order of DPs promoting cell proliferation was DH > DNL > DCL > DFH > DO. Notably, DH, DNL, DCL, and DFH exhibited the strongest proliferative effects at doses of 400 μg/mL, which were 2.09, 1.71, 1.59, and 1.57 times higher than those of the control. These findings elucidate that DPs are biocompatible and provide a basis for subsequent applications of DPs.

#### 3.4.2. Effects of DPs on NO Production

NO is an important effector molecule for non-specific host defense against tumor and microbial invasion [22]. The production of large amounts of NO in an inflammatory environment can cause cytotoxicity and disrupt the normal function of NO as a signaling molecule in the nervous system [23]. Thus, studies have shown that the inhibition of NO release can attenuate cellular damage as a result of inflammatory responses. As shown in Figure 4B, when RAW 264.7 was activated by LPS, the amount of NO released from the LPS group was significantly increased compared to the control group (*p* < 0.05), indicating that inflammation modeling was successful. Meanwhile, NO production decreased with increasing DO, DNL, and DCL concentrations (10–200 μg/mL). However, with the increase in DH and DFH concentrations, NO production decreased firstly and then increased. It is worth mentioning that the best inhibitory activity of DPs was observed at 50 μg/mL; after DO, DH, DNL, DFH, DCL, and DF treatment, NO production was 0.17, 0.05, 0.17, 0.11, and 0.06 for the LPS group, respectively. NO production was inhibited by DPs in the order DH > DCL > DFH > DO > DNL (50 μg/mL). The results suggest that DPs can reduce cell damage and are good mitigators of oxidative stress, restoring NO to its normal role as a signaling molecule. It is worth noting that DH has the best anti-inflammatory activity, which is consistent with the highest proportion of →4)Man*p*(1→ and →4)Glc*p*(1→ glycosidic bonds in DH polysaccharides as shown in Table 4. A previous study found that the 1 →4 and 1 →6 bonds are particularly flexible unit structures in polysaccharides and can enhance activity by increasing the favorable entropy of the solution [24]. Furthermore, DH is the most precious species in Dendrobium, and the results in this study also support its clinical usage. There are also studies that found that *Dendrobium nobile* Lindl. (DNL) polysaccharides (12.5–200 μg/mL) inhibited NO release in a dose-dependent manner [25]. Therefore, it can be inferred that the inhibition of NO production is a key factor in activating the anti-inflammatory activity of DPs.

NO release was involved in physiological and pathological processes and may also be involved in the regulation of apoptosis by altering. Therefore, the effect of DPs on macrophage RAW 264.7 inflammation needs to be further explored by measuring apoptotic activity [26,27].

#### 3.4.3. Effects of DPs on Apoptosis

**Figure 5:**
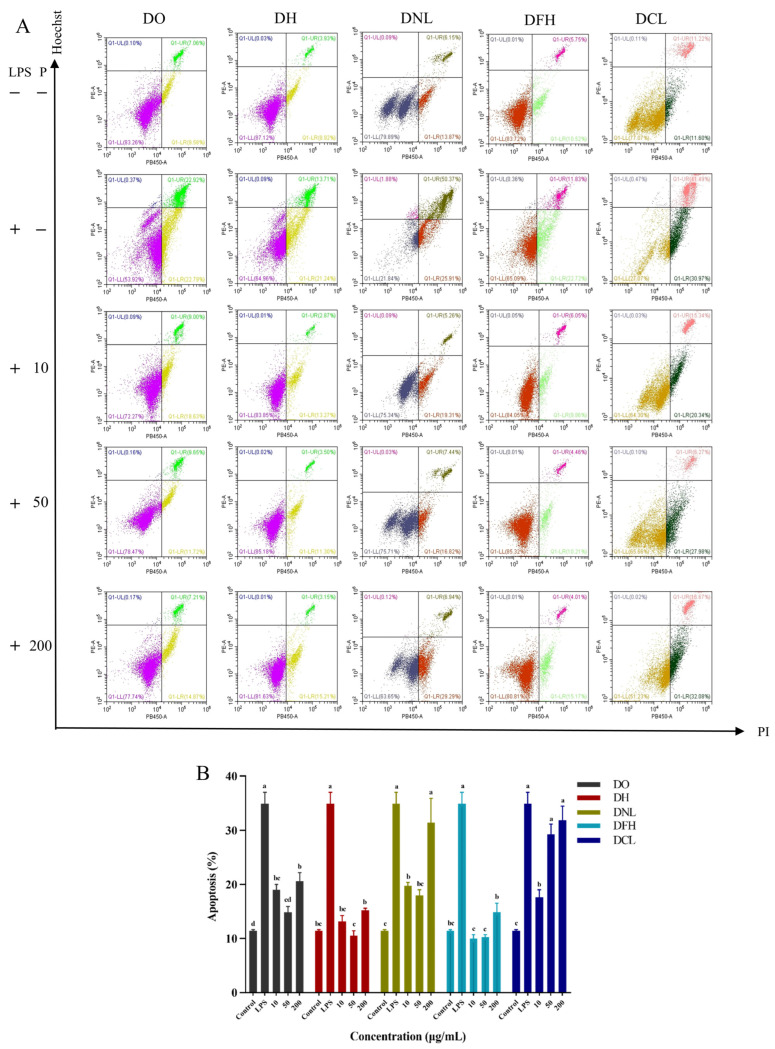
Apoptosis was detected by flow cytometry. Contour diagram of the FITC–AnnexinV/PI flow cytometry of RAW 264.7 cells (**A**). Calculation of apoptotic cells according to (**A**). Data are presented as the mean ± S.D (*n* = 3) (**B**). a–d—Different letters indicate significant differences (*p* < 0.05).

Apoptosis is an important mechanism for stabilizing the intracellular environment and plays an important role as a defense system in clearing damaged, infected, or mutated cells [28]. To assess the effect of DPs on apoptosis in RAW 264.7 cells, flow cytometry analysis was performed to detect apoptosis after membrane-bound protein V-FITC/PI double-staining. It was found that the LPS-induced inflammatory model not only exhibited inflammation but also induced apoptosis. As shown in Figure 5A,B, the number of apoptotic cells was significantly increased in the LPS group compared with the control group, indicating significant apoptosis, suggesting that the inflammation modeling was successful. After treatment with different concentrations of DPs (10–200 μg/mL), DPs showed an inhibition of apoptosis levels. As the concentration of DPs first increased, the inhibition of apoptosis levels occurred at a low concentration (10–50 μg/mL), while the apoptosis levels increased at 200 μg/mL, nearly reaching those of the LPS group. At a 50 μg/mL dose of DO, DH, DNL, DFH, and DCL treatments, apoptosis levels were 0.43, 0.30, 0.51, 0.29, and 0.84 for the LPS group, and DPs decreased apoptosis levels in the order of DFH > DH > DO > DNL > DCL. Thus, we can see that apoptosis is also triggered along with inflammation in the LPS-induced RAW 264.7 macrophage cell model, which can also be partially reversed by DP administration. A previous study found that DO polysaccharides (50 μg/mL) could reduce LPS-induced apoptosis [29]. Furthermore, DO polysaccharides could also ameliorate Uropathogenic *Escherichia coli* (UPEC)-induced apoptosis in macrophage cells [30].

### 3.5. Correlation Analysis of Structure and Anti-Inflammatory Activity

In order to further evaluate the relationship between the structure and anti-inflammatory activity of Dendrobium polysaccharides, gray correlation analysis was performed using SPSSPRO. The correlation coefficient was calculated by gray relational analysis, and the range was 0–1, with an increase in the value indicating a higher correlation. As shown in Table 5, the gray correlation rank was calculated and ranking performed according to the gray correlation coefficient, with r > 0.70 selected as the specific structural feature that mainly affects activity in this study. The most important factor, which showed the highest correlation coefficient of 0.765, is glycosidic bond types, →4)Man*p*(1→/→4)Glc*p*(1→. Then, the molecular weight of DPs ranged from 3.343 ± 0.061 × 10^5^ Da to 13.540 ± 0.033 × 10^5^ Da (Peak 1) with a correlation coefficient of 0.748. The results confirm that Dendrobium polysaccharides with molecular weights of 3.343 ± 0.061 × 10^5^ Da—13.540 ± 0.033 × 10^5^ Da, and a backbone of →4)Man*p*(1→ and →4)Glc*p*(1→, especially at high proportions, showed strong correlation with anti-inflammatory activity. Based on this result, it is indicated that the quality evaluation of Dendrobium in clinical applications should investigate the molecular weight and the composition of the glycoside bond of the backbone to ensure the consistency of curative effects. These results also have significance in elucidating the anti-inflammatory activity of Dendrobium polysaccharides.

## 4. Conclusions

In this study, the structural characteristics of polysaccharides from five Dendrobium species (including DH, DO, DNL, DFH, and DCL) were interpreted and compared, including their Mw distribution, monosaccharide composition, and glycosidic bond type. The types and proportions of glycosidic bonds reveal additional information about the differences in polysaccharides from different species of Dendrobium. According to the structure and anti-inflammatory activity of polysaccharides, it was found that polysaccharides with →4)Man*p*(1→ and →4)Glc*p*(1→ as a backbone and a molecular weight between 3.343 ± 0.061 × 10^5^ Da and 13.540 ± 0.033 × 10^5^ Da exhibited significant anti-inflammatory activity. These results suggest that for future pharmaceutical or medical applications, components with this specific structure could be prioritized for development as anti-inflammatory drugs, thereby providing a foundation for the advancement of anti-inflammatory therapeutics. Furthermore, the results also showed that the comprehensive structural characteristics of polysaccharides should be considered when evaluating the activity of polysaccharides.

## Figures and Tables

**Table 1:** Studied *Dendrobium* spp.

No.	Species	Codes	Origins
1	*Dendrobium officinale* Kimura et Migo	DO1	Anhui Province
2		DO2	Anhui Province
3		DO3	Yunnan Province
4		DO4	Yunnan Province
5	*Dendrobium huoshanense* C. Z. Tang & S. J. Cheng	DH1	Anhui Province
6		DH2	Anhui Province
7		DH3	Anhui Province
8	*Dendrobium nobile* Lindl.	DNL1	Yunnan Province
9		DNL2	Guizhou Province
10		DNL3	Yunnan Province
11	*Dendrobium fimbriatum* Hook.	DFH1	Yunnan Province
12		DFH2	Yunnan Province
13	*Dendrobium chrysanthum* Lindl.	DCL1	Yunnan Province
14		DCL2	Yunnan Province

**Table 2:** Molecular weight (Mw) and polydispersity index (PDI) of polysaccharide fractions from *Dendrobium* species.

	Peak 1		Peak 2	
	Mw (×10^5^ Da)	PDI	Mw (×10^5^ Da)	PDI
DO1	8.905 ± 0.039 ^b^	1.194 ± 0.057	2.119 ± 0.026 ^b^	1.047 ± 0.042
DO2	12.330 ± 0.043 ^a^	1.142 ± 0.049	2.829 ± 0.033 ^a^	1.068 ± 0.029
DO3	5.698 ± 0.046 ^c^	1.208 ± 0.053	1.539 ± 0.049 ^c^	1.049 ± 0.054
DO4	5.016 ± 0.026 ^d^	1.271 ± 0.024	1.236 ± 0.033 ^d^	1.133 ± 0.020
Average	7.987 ± 3.035 ^AB^	1.204 ± 0.063	1.204 ± 0.063 ^AB^	1.074 ± 0.049
DH1	7.978 ± 0.044 ^a^	1.197 ± 0.061	1.727 ± 0.051 ^a^	1.078 ± 0.057
DH2	4.546 ± 0.055 ^c^	1.121 ± 0.063	1.553 ± 0.058 ^b^	1.071 ± 0.070
DH3	4.727 ± 0.024 ^b^	1.156 ± 0.033	1.699 ± 0.032 ^a^	1.094 ± 0.041
Average	5.750 ± 1.673 ^AB^	1.158 ± 0.057	1.660 ± 0.091 ^AB^	1.081 ± 0.051
DNL1	5.594 ± 0.027 ^b^	1.287 ± 0.058	1.579 ± 0.030 ^a^	1.053 ± 0.048
DNL2	4.810 ± 0.043 ^c^	1.226 ± 0.065	1.274 ± 0.027 ^b^	1.120 ± 0.081
DNL3	5.760 ± 0.053 ^a^	1.172 ± 0.029	1.584 ± 0.025 ^a^	1.166 ± 0.037
Average	5.388 ± 0.441 ^AB^	1.228 ± 0.068	1.479 ± 0.156 ^AB^	1.113 ± 0.071
DFH1	6.514 ± 0.047 ^b^	1.286 ± 0.039	1.640 ± 0.041 ^b^	1.031 ± 0.038
DFH2	13.540 ± 0.033 ^a^	1.085 ± 0.046	4.960 ± 0.021 ^a^	1.642 ± 0.030
Average	10.027 ± 3.848 ^A^	1.186 ± 0.117	3.300 ± 1.819 ^A^	1.337 ± 0.336
DCL1	3.364 ± 0.058 ^a^	1.174 ± 0.037	0.855 ± 0.047 ^a^	1.123 ± 0.087
DCL2	3.343 ± 0.061 ^a^	1.622 ± 0.039	0.563 ± 0.049 ^b^	1.117 ± 0.072
Average	3.354 ± 0.054 ^B^	1.398 ± 0.248	0.709 ± 0.166 ^B^	1.120 ± 0.071

^a–d^ Different superscript letters indicate significant differences in the same species (*p* < 0.05). ^A,B^ Different superscript letters indicate differences between the two species (*p* < 0.05). Polydispersity index (PDI) values indicate the heterogeneity of molecular weight distributions.

**Table 3:** The compositional monosaccharides of polysaccharides in different species of *Dendrobium*.

	Ara	Xyl	Man	Glc	Gal	Am/Ag	Am/Ag Range
DO1	0.41	0.36	122.42	31.84	1.00 ^a^	3.84	2.23–4.22
DO2	0.35	0.44	121.13	28.72	1.00	4.22	
DO3	0.47	0.48	333.50	127.77	1.00	2.61	
DO4	0.83	0.89	339.90	152.69	1.00	2.23	
Average	0.52 ± 0.22	0.54 ± 0.24	229.24 ± 124.12	85.26 ± 64.30	1.00	3.23 ± 0.96	
DH1	0.58	0.00	348.47	102.61	1.00	3.40	3.40–4.49
DH2	0.69	0.00	485.41	137.17	1.00	3.54	
DH3	0.73	0.70	402.77	89.64	1.00	4.49	
Average	0.67 ± 0.08	0.23 ± 0.40	412.22 ± 68.96	109.81 ± 24.57	1.00	3.81 ± 0.59	
DNL1	0.40	0.00	289.32	105.23	1.00	2.75	2.75–4.76
DNL2	0.63	0.78	294.01	71.21	1.00	4.13	
DNL3	0.71	0.87	274.61	57.74	1.00	4.76	
Average	0.58 ± 0.16	0.55 ± 0.48	285.98 ± 10.12	78.06 ± 24.47	1.00	3.88 ± 1.03	
DFH1	0.81	0.61	97.35	232.99	1.00	0.42	0.42–0.48
DFH2	0.84	0.81	111.79	232.23	1.00	0.48	
Average	0.83 ± 0.02	0.71 ± 0.14	104.57 ± 10.21	232.61 ± 0.54	1.00	0.45 ± 0.04	
DCL1	0.70	0.77	44.89	52.15	1.00	0.86	0.76–0.86
DCL2	0.49	0.42	30.38	40.03	1.00	0.76	
Average	0.60 ± 0.15	0.60 ± 0.25	37.64 ± 10.26	46.09 ± 8.57	1.00	0.81 ± 0.07	

^a^ The proportion of each monosaccharide in the composition of samples is normalized to Gal. Am/Ag represents the ratio of Man to Glc in polysaccharides.

**Table 4:** The glycosidic bond types of polysaccharides in different species of *Dendrobium*.

Peak	1	2	3	4	5	6	7	8		
Glycosidic Bonds	Ara*f* (1→	→5) Ara*f* (1→	Glc*p* (1→	→3,5) Ara*f* (1→	→4) Man*p* (1→	→4) Glc*p* (1→	→4,6) Man*p* (1→	→4,6) Glc*p* (1→	→4)Man*p*(1→/→4)Glc*p*(1→	→4)Man*p* (1→/→4) Glc*p*(1→ Average Value
DO1	0.03	0.79	0.99	1.00 ^a^	10.09	3.63	0.05	0.03	2.78	2.85
DO2	/ ^b^	1.41	0.78	1.00	10.75	4.11	0.04	0.04	2.62	
DO3	0.03	0.66	0.65	1.00	11.18	3.43	0.85	0.27	3.26	
DO4	0.03	3.08	0.56	1.00	10.26	3.73	0.80	0.23	2.75	
DH1	0.06	0.12	0.58	1.00	20.72	7.23	0.10	0.09	2.87	2.92
DH2	0.14	0.01	0.66	1.00	28.61	9.89	0.20	0.16	2.89	
DH3	0.41	11.05	2.85	1.00	46.11	15.31	0.87	0.28	3.01	
DNL1	0.03	1.35	0.94	1.00	8.61	5.94	0.24	0.20	1.45	1.50
DNL2	0.02	0.48	0.80	1.00	9.75	7.05	0.19	0.19	1.38	
DNL3	0.02	1.93	0.99	1.00	8.62	5.16	0.06	0.09	1.67	
DFH1	0.03	1.78	0.91	1.00	7.95	4.66	0.05	0.08	1.71	1.45
DFH2	/	0.20	1.11	1.00	7.64	6.43	0.03	0.06	1.19	
DCL1	0.48	0.75	1.26	1.00	9.58	9.43	0.01	0.03	1.02	1.05
DCL2	0.01	0.69	1.55	1.00	11.43	10.49	0.03	0.07	1.09	

^a^ The proportion of each glycosidic bond of samples is normalized to →3,5)Ara*f*(1→. ^b^ Undetected.

**Table 5:** Gray relational analysis.

NO Production					
Molecular Weight Distribution		Monosaccharide Composition		Glycosidic Linkage Types	
Peaks	Correlation Coefficient	Monosaccharide	Correlation Coefficient	Glycosidic Bond	Correlation Coefficient
Peak 1	0.748	Xyl	0.746	→4)Man*p*(1→/→4)Glc*p*(1→	0.765
Peak 2	0.682	Gal	0.731	→4,6)Glc*p*(1→	0.740
		Am/Ag	0.724	→3,5)Ara*f*(1→	0.731
		Man	0.671	Glc*p*(1→	0.638
		Ara	0.667	→5)Ara*f*(1→	0.637
		Glc	0.632	→4)Man*p*(1→	0.593
				→4)Glc*p*(1→	0.585
				→4,6)Man*p*(1→	0.568
				Ara*f*(1→	0.427

Am/Ag represents the ratio of Man to Glc in polysaccharides.

## Data Availability

Data are contained within the article.
